# *VacA* and *CagA* Status as Biomarker of Two Opposite End Outcomes of *Helicobacter pylori* Infection (Gastric Cancer and Duodenal Ulcer) in a Moroccan Population

**DOI:** 10.1371/journal.pone.0170616

**Published:** 2017-01-26

**Authors:** Mounia El Khadir, Samia Alaoui Boukhris, Dafr-Allah Benajah, Karima El Rhazi, Sidi Adil Ibrahimi, Mohamed El Abkari, Taoufiq Harmouch, Chakib Nejjari, Mustapha Mahmoud, Mohamed Benlemlih, Bahia Bennani

**Affiliations:** 1 Laboratoire de Microbiologie et de Biologie Moléculaire, Equipe Micro-organismes, Génomique et Facteurs Oncogènes, Faculté de Médecine et de Pharmacie de Fès (FMPF), Université Sidi Mohammed Ben Abdellah (USMBA), Fez, Morocco; 2 Laboratoire de Biotechnologie, Faculté des Sciences Dhar El Mehraz, USMBA, Fez, Morocco; 3 Service d’Hépato Gastro-entérologie, CHU Hassan II de Fès, Equipe Maladies de l’Appareil Digestif, FMPF, Fez, Morocco; 4 Laboratoire de Pathologie Humaine, Biomédecine et Environnement, FMPF, USMBA, Fez, Morocco; 5 Laboratoire d’Epidémiologie et de Recherche Clinique, FMPF, USMBA, Fez, Morocco; 6 Service d’Anatomie Pathologique CHU Hassan II, Fez, Morocco; 7 Service de Bactériologie CHU Hassan II, Fez, Morocco; Vanderbilt University Medical Center, UNITED STATES

## Abstract

*Helicobacter pylori* (*H*. *pylori*) infection induces inflammation of the gastric mucosa, which may progress to precancerous lesions leading to gastric cancer. Pathological determinism is associated to some virulence genes of the bacterium, notably the *vacA* and *cagA* genes. The present study aimed to determine the *H*. *pylori* genotypes distribution and their association with sex, age and gastric diseases in a Moroccan population. Gastric biopsy was taken from 1079 consenting patients. The specimens were processed by PCR to identify *H*. *pylori* and to determine the genotypic profile by PCR characterizing *vacA s*, *vacA m* and *vacA i* regions directly from biopsies *H*. *pylori* positives. *VacA* genotyping revealed the predominance of *vacA m2* (53.2%), *vacA s2* (52.9%) and *vacA i2* (52%). The most virulent *vacA* alleles (s1, i1 and m1) are more predominant in men (47.3%, 41.9% and 46.1% respectively) than in women (38.3%, 33.3% and 37% respectively). However, the association between *vacA* genotypes and age did not reach a statistical significant value. Logistic regression analysis results show that *vacA i1m1* and *vacA i1m2* genotypes were strongly associated with the risk of GC, the Odds Ratio (95% confidence interval) was 29.73 [5.08–173.73] and 9.17 [2.06–40.82] respectively, while vacA*s1/cagA*+ seems to be a risk factor for DU since it is inversely associated with GC (OR was 0.13 [0.02–0.75]. The results of this study suggest that *vacA i1* genotype independently to *vacAm* status may be of a clinical usefulness and will help to identify patients at a high risk of GC development.

## Introduction

Gastric cancer is the third common cause of cancer mortality in the world. Multiple epidemiological studies have documented an increased incidence of gastric cancer with increased prevalence of *H*. *pylori* infection. In Morocco, as in other African and South East Asian countries, there is a paradox between the high prevalence of *H*. *pylori* infection (59.7%) [1], and low incidence of gastric cancer (5.6%) [2]. The difference of the geographic distribution of *H*. *pylori* infection and gastric cancer incidence suggests the presence of the determining factors which could influence the interaction between pathogen and host. Those factors include: human genetic polymorphism, environmental influences and the high genomic diversity of *H*. *pylori*. Overall, the genetic diversity of the bacteria occurs mainly in the virulence factors such as the cytotoxin-associated gene (*cagA*) and the vacuolating cytotoxin (*vac*A). CagA protein is a 120- to 140-kDa protein that is translocated into host cells by the type IV cag secretion system after bacterial attachment, altering thereafter the cell-signaling mechanisms in gastric cells. VacA protein is a cytotoxin inducing vacuolation of the epithelial cell; the gene is present in all strains but only some of them have a vacuolating activity. This variation is attributed to variations in *vacA* gene structures within the three regions: the signal sequence region (*s*-region) (s1 or s2), mid-region (*m*-region) (m1 or m2) and the intermediate-region (*i*-region) (i1, i2 or i3) [3–5]. The polymorphism of these two virulent factors has been the subject of numerous investigations [6–9].

In Morocco, we previously studied the status of *cagA* and the genotypes of *vacA-s*, -*m* regions. The preliminary results show the predominance of *vacA s2m2* which was significantly associated with gastritis while the *vacA s1m1* was significantly associated with peptic ulcer diseases[10]. Although, there was no significant difference in the prevalence of these two genotypes in gastric cancer [10]. When the *H*. *pylori* genotypes were correlated to histological lesions, significant associations between gastric cancer, *cagA+* and *vacA m1* genotypes were obtained in older patients [1]. Indeed, these preliminary results need to be confirmed in larger sampling to better characterize isolates that may lead to severe diseases and by exploring another *vacA* intermediate region (*vacA i*) described as strong marker of *H*. *pylori* associated disease among the *vacA* alleles [5]. To the best of our knowledge, this is the first study reported in Morocco and in the North Africa that aimed to evaluate the genetic polymorphism among *H*. *pylori* strains isolated from Moroccan patients on the basis of their *vacA* genotypes (s, m, and i) and *cagA* status. It aimed also to find the association of the *vacA* genotypes with sex, age and gastric diseases.

## Materials and Methods

### Patients and sampling

This study was conducted between May 2009 and January 2015. The biopsies of 801 patients, previously characterized on the basis of *vacA-s*, -*m* regions and *cagA* status were used to determine *vacA i* genotype and 278 patients were prospectively recruited and added to the existing cases to be analyzed.The total of consenting patients aged 15 years or more, who were attending the gastroenterology department of Hospital University (CHU) Hassan II of Fez, Morocco, and who had undergone endoscopy for the diagnosis of abdominal pain or discomfort were included in this study. However, patients aged less than 15 years or who were on medications (antibiotics, proton pump inhibitors) for the last 3 months and also pregnant or nursing women were excluded. The recruited patients have an average age of 49.30 ± 16.29 years, ranging from 15 to 99 years, and had a personal interview, where they were asked about individual characteristics. A total of three biopsies were collected from each patient during the endoscopy: one biopsy from the antrum which was directly used for molecular detection of *H*. *pylori*, *cagA* status and *vacA* genotyping by polymerase chain reaction (PCR). The other biopsies (one from antrum and the other from corpus) were examined independently by an experimented anatomopathologists.

All participants were informed about the study objectives, methods, confidentiality, and potential outcomes and they provided written informed consent for their participation. Also, parental consent was obtained on the behalf of the participants under the age of 18. In the case of illiterate or semi-literate patients, the written consent was read to them by the interviewer.This study was approved by the Institutional Review Board of the Hassan II University Hospital of Fez, Morocco.

### DNA extraction

Using the protocol previously described (10), DNA was extracted from the gastric antrum biopsy specimens and stored at -20°C until molecular analysis.

### Polymerase chain reaction (PCR)

*H*. *pylori* was detected in biopsies by PCR using *glmM* primers as described previously [11]. Positive Samples were subjected to multiplex PCR in order to determine the *cagA status*, *vacA s* and *vacA m* subtypes [12,13] and also to simple PCR to determine the *vacA i* polymorphisms using specific primers as previously described [5].

Samples with non identified genotypes (*vacA s* and *m* alleles*)* in multiplex PCR were subjected to PCR reactions using the same primer sets but in single reactions. Likewise, all non amplified *vacA i* cases were subjected to another PCR using the sense primer performed by Ferreira [14] and two antisense primers designed by Rhead [5].

The *cagA* status has been verified using two primer sets as previously described [6] in order to increase the sensitivity of detection. In fact, *cagA* was considered positive if it shows positive results with one of the three primers pairs.

Negative and positive controls were used for each reaction.

### Statistical analysis

The statistical analysis was done using SPSS (Statistical product and services solutions, version 20, SPSS Inc. Chicago, Illinois, USA) software. It consisted primarily to describe the study population, for this, the results were presented as mean ± standard deviation for quantitative variables and number (percentage) for qualitative variables. Then, an univariate analysis was performed to establish all associations between clinical diseases, age, gender, *H*. *pylori* infection, and *H*. *pylori* genotypes (Only samples with single-strain infection (identified on the basis of vacuolating cytotoxin gene (*vacA* alleles) and complete *vacA* alleles were considered), Chi-square or Fisher’s exact tests were applied to establish all statistically significant associations; A p value < 0.05 was considered as significant. The multivariate analysis was performed to highlight the most incriminated factors in the occurrence of GC using simple logistic regression analysis. All variables with p ≤ 0.20 were included in the initial model. The final model was obtained using a stepwise elimination method to identify potential independent factor(s) associated with GC. The results were expressed as odds ratio (OR), 95% confidence intervals (CIs) and p-values.

## Results

A total of 1079 patients from urban and rural areas of north center of Morocco and consulting for abdominal pain or discomfort were included in this study. They were 519 women (48.1%) and 560 men (51.9%). The clinical exam shows that 738 patients were with chronic gastritis, 260 with peptic ulcer disease (PUD), 119 with gastric ulcer [GU], 133 duodenal ulcers [DU], 8 with GU and DU and 81 patients were with gastric cancer (GC) [adenocarcinoma or gastric lymphoma]. All GC cases were confirmed by histological exam.

PCR results show that *H*. *pylori* infection rate in the studied population is 59.4% (641/1079). *VacA* and *cagA* genes were amplified in 99.53% (638/641) and 61.2% (392/641) cases respectively. The *vacA s-*, *i-* and *m-* regions were amplified in 99.06% (635/641), 90.01% (577/641) and 95% (609/641) of the cases respectively. The determined rate of *vacA m2*, *s2* and *i2* alleles was 53.2% (324/609), 52.9% (336/635) and 52% (300/577) respectively “Table 1”.

**Table 1:** Correlation between *vacA* mosaicism and *cagA* status of *H*.*pylori* strains and their distribution according to the gender and to the age.

	Gender N (%)			Age N (%)			*cagA* N (%)			Total N (%)
genotypes	Male	Female	p-value *	< 50 years	≥ 50 years	p-value *	+	-	p-value*	
***vacA***										
*s1*	157 (47.3)	116 (38.3)	0.005	151 (45.8)	122 (40)	0.11				273 (43)
*s2*	155 (46.7)	181 (59.7)		164 (49.7)	172 (56.4)					336 (52.9)
*s1s2*	20 (6)	6 (2)		15 (4.5)	11 (3.6)					26 (4.1)
*i1*	125 (41.9)	93 (33.3)	0.020	108 (35.5)	110 (40.3)	0.53				218 (37.8)
*i2*	141 (47.3)	159 (57)		157 (51.6)	143 (52.4)					300 (52)
*i1i2*	32 (10.7)	27 (9.7)		39 (12.8)	20 (7.3)					59 (10.2)
*m1*	146 (46.1)	108 (37)	0.009	137 (42.7)	117 (40.6)	0.518				254 (41.7)
*m2*	151 (47.6)	173 (59.2)		166 (51.7)	158 (54.9)					324 (53.2)
*m1m2*	20 (6.3)	11 (3.8)		18 (5.6)	13 (4.5)					31 (5.1)
*s1m1i1*	82 (24.4)	55 (18)	0.009#	73 (21.9)	64 (20.8)	0.652#	118 (86.1)	19 (13.9)	<0.001#	137 (21.4)
*s2i2m2*	91 (27.1)	120 (39.3)		108 (32.4)	103 (33.4)		84 (39.8)	127 (60.2)		211 (32.9)
*s1i2m1*	9 (2.7)	8 (2.6)		6 (1.8)	11 (3.6)		14 (82.4)	3 (17.6)		17 (2.7)
*s1i1m2*	10 (3)	6 (2)		11 (3.3)	5 (1.6)		14 (87.5)	2 (12.5)		16 (2.5)
*s1i2m2*	19 (5.7)	19 (6.2)		21 (6.3)	17 (5.5)		34 (89.5)	4 (10.5)		38 (5.9)
*s2i1m1*	10 (3)	12 (3.9)		9 (2.7)	13 (4.2)		14 (63.6)	8 (36.4)		22 (3.4)
*s2i2m1*	6 (1.8)	4 (1.3)		6 (1.8)	4 (1.3)		3 (30)	7 (70)		10 (1.6)
*s2i1m2*	12 (3.6)	13 (4.3)		8 (2.4)	17 (5.5)		11 (44)	14 (56)		25 (3.9)
Inc vac	40 (11.9)	31 (10.2)		28 (8.4)	43 (14)		28 (39.4)	43 (60.6)		71 (11.1)
Vac MI	55 (16.4)	36 (11.8)		61 (18.3)	30 (9.7)		71 (78)	20 (22)		91 (14.2)
VacA NT	2 (0.6)	1 (0.3)		2 (0.6)	1 (0.3)		1 (33.3)	2 (66.7)		3 (0.5)
***cagA***										
+	212 (63.1)	180 (59)	0.290	224 (67.3)	168 (54.5)	0.001				392 (61.2)
-	124 (36.9)	125 (41)		109 (32.7)	140 (249)					249 (38.8)

Inc vacA: Incomplet VacA; Vac MI: Multiple infection; VacA NT: VacA not genotyped.

* p-value was considered only in cases with single-strain infection (identified on the basis of vacuolating cytotoxin gene (*vacA* alleles)

# Only the most predominant genotypes of the simple infection with complete *vacA* genotype (*vacA s2i2m2*, *vacA s1i1m1* and the other combination allelic of *vacA*) were considered in the statistical analysis.

The distribution of the intermediate region of *vacA* among the s and m regions was studied in this series and reveals that 89% and 89.4% of *vacA s1m1* and *vacA s2m2* strains were *vacA i1* and *vacA i2* respectively. Also, our results show that 70.4% of the *vacA s1m2* strains were *vacA i2* “Table 2”.

**Table 2:** Association between *H*. *pylori vacA* alleles in studied isolates from Moroccan patients with single-strains infections.

		Intermediate region of *vacA* N (%)		
	Subtype	*i1*	*i2*	p-value
*vacA*	*s1m1*	137 (89)	17 (11)	<0.001
	*s2m2*	25 (10.6)	211 (89.4)	
	*s1m2*	16 (29.6)	38 (70.4)	
	*s2m1*	22 (68.8)	10 (31.2)	

Multiple infections (presence of the 2 alleles of the same *vacA* region (s, m or i)) and incomplete *vacA* (lack of the two alleles of the same *vacA* region) were detected in 14.2% (91) and 11.1% (71) respectively of the 641 subjects evaluated. Excluding these 2 profiles (incomplete *vacA* and multiple infection), *vacA* genotype distribution revealed a large predominance of *vacA s2i2m2* with rates of 44.3% (211/476) while *vacA s1i1m1*, *vacA s1i2m2*, *vacA s2i1m2* and *vacA s2i1m1* were detected in 28.8% (137/476), 8% (38/476), 5.3% (25/476) and 4.6% (22/476) respectively. The rate of each genotype is reported in Table 1.

The correlation between *cagA* and *vacA* mosaic genotypes in only samples with simple infection and with complete *vacA* genotypes (1: *vacA s1i1m1*; 2: *vacA s2i2m2*; 3: Other less prevalent genotypes of *vacA*) was studied. The results show a significant association between *vacA s1i1m1* and *cagA+* and between *vacA s2i2m2* and *cagA-* (p < 0.001) “Table 1”.

Correlation between age and sex with *vacA* genotypes and *cagA* status was also tested in two groups: the group 1 includes young patients aged less than 50 years while the group 2 includes those with 50 years and older. The statistical analysis revealed that the most virulent *vacA* alleles (s1, i1 and m1) are more predominant in men (47.3%, 41.9% and 46.1% respectively) than in women (38.3%, 33.3% and 37% respectively) (p = 0.005, 0.020 and 0.009 respectively), while *cagA*+ is more frequent in age group 1 (67.3%) than in age group 2 (54.5%) (p = 0.001) “Table 1”. However, the association between *vacA* genotypes and age did not reach a statistical significant value.

The distribution of the pathologies according to the patients gender, age, *H*. *pylori* infection, and *H*. *pylori* genotypes was also studied. The results show that GC, GU, and DU were predominant in men (63%, 69.7% and 73.7% cases respectively) while a high prevalence of gastritis was found among women (56.5%). According to the PCR results, *H*. *pylori* prevalence is high in all gastric disease with predominance in patients with GC (92.6%). The association of each of the three *vacA* regions with clinical outcomes shows that *vacA s2*, *vacA i2* and *vacA m2* were predominant in gastritis cases (59.5%, 59.9% and 58.7.5% respectively). *VacA s1* was present in 57.4% of patients with DU (p <0.001), *vacA i1* and *vacA m1* were present in GC patients with rates of 73.3% and 65.7% respectively (p <0.001) (Table 3).

**Table 3:** Distribution of Demographic and risk factors of the gastro duodenal diseases.

		N (%)				
		Gastritis	GU	DU	GC	P-value
	Positive	399 (54.1)	67 (56.3)	94 (70.7)	75 (92.6)	< 0.001
***H*. *pylori***	Negative	339 (45.9)	52 (43.7)	39 (29.3)	6 (7.4)	
***cagA***	positive	229 (57.4)	44 (65.7)	74 (78.7)	42 (56)	0.001
	negative	170 (42.6)	23 (34.3)	20 (21.3)	33 (44)	
***vacA s***	*s1*	145 (36.7)	37 (55.2)	54 (57.4)	32 (43.8)	< 0.001
	*s2*	235 (59.5)	26 (38.8)	34 (36.2)	40 (54.8)	
	*s1s2*	15 (3.8)	4 (6)	6 (6.4)	1 (1.4)	
***vacA i***	*i1*	112 (31.4)	24 (38.7)	35 (37.6)	44 (73.3)	< 0.001
	*i2*	214 (59.9)	34 (54.8)	40 (43)	12 (20)	
	*i1i2*	31 (8.7)	4 (6.5)	18 (19.4)	4 (6.7)	
***vacA m***	*m1*	137 (36.1)	27 (42.9)	40 (44.4)	46 (65.7)	< 0.001
	*m2*	223 (58.7)	31 (49.2)	45 (50)	24 (34.3)	
	*m1m2*	20 (5.3)	5 (7.9)	5 (5.6)	0	
***vacA* mosaic**	*s1i1m1*	66 (16.5)	18 (26.9)	27 (28.7)	24 (32)	<0.001
	*s1i2m1*	12 (3)	3 (4.5)	2 (2.1)	0	
	*s1i1m2*	13 (3.3)	0 (0)	2 (2.1)	1 (1.3)	
	*s1i2m2*	21 (5.3)	8 (11.9)	8 (8.5)	1 (1.3)	
	*s2i1m1*	11 (2.8)	1 (1.5)	0	9 (12)	
	*s2i2m1*	7 (1.8)	1 (1.5)	0	2 (2.7)	
	*s2i1m2*	13 (3.3)	1 (1.5)	3 (3.2)	8 (10.7)	
	*s2i2m2*	159 (39.8)	18 (26.9)	26 (27.7)	8 (10.7)	
	Inc VacA	44 (11)	7 (10.4)	3 (3.2)	16 (21.3)	
	Multiple infection	51 (12.8)	10 (14.9)	23 (24.5)	5 (6.7)	
	Not genotype	2 (0.5)	0	0	1 (1.3)	
**Sex**	Male	321 (43.5)	83 (69.7)	98 (73.7)	51 (63)	< 0.001
	Female	417 (56.5)	36 (30.3)	35 (26.3)	30 (37)	
**Age**	< 50 years	362 (49.1)	48 (40.3)	73 (54.9)	30 (37)	0.023
	≥ 50 years	376 (50.9)	71 (59.7)	60 (45.1)	**51 (63)**	

To find out a possible association between *vacA* alleles (s, m and i) combination and the studied diseases, only the most predominant genotypes of the simple infections (*vacA s2i2m2*, *vacA s1i1m1* and the other combination allelic of *vacA*) were considered in the statistical analysis and the results show that the rate of *vacA s1i1m1* in GC patients was higher than the other *vacA* combinations. Also, a high prevalence of *vacA s2i2m2* in gastritis patients was noted. While according to *cagA* gene status, *cagA+* was higher in DU cases (78.7%) than the other pathologies (Table 3).

To evaluate the effect of each genotype and demographic characteristics on the occurrence of GC, we have considered the DU cases as reference control. Initially, univariate analysis was done and is shown in Table 4 and Table 5.

**Table 4:** Distribution of demographic and *vacA* alleles (single and associated) of *H*. *pylori* in duodenal ulcers and gastric cancer cases.

		N (%)							
		DU	GC	p-value			DU	GC	p-value
**H. pylori**	+	94 (70.7)	75 (92.6)	>0.001	***vacA si***	*s1i1*	30 (42.3)	25 (46.3)	> 0.001
	-	39 (29.3)	6 (7.4)			*s1i2*	10 (14.1)	1 (1.9)	
**Sexe**	Male	98 (73.7)	51 (63)	0.098		*s2i1*	4 (5.6)	18 (33.3)	
	Female	35 (26.3)	30 (37)			*s2i2*	27 (38)	10 (18.5)	
**Age**	< 50 years	73 (54.9)	30 (37)	0.011	***vacA im***	*i1m1*	27 (38.6)	33 (61.1)	0.002
	≥ 50 years	60 (45.1)	51 (63)			*i2m1*	2 (2.9)	2 (3.7)	
***vacA s***	*s1*	54 (61.4)	32 (44.4)	0.033		*i1m2*	5 (7.1)	9 (16.7)	
	*s2*	34 (38.6)	40 (55.6)			*i2m2*	36 (51.4)	10 (18.5)	
***vacA i***	*i1*	35 (46.7)	44 (78.6)	>0.001	***vacA sm***	*s1m1*	39 (47.6)	30 (43.5)	> 0.001
	*i2*	40 (53.3)	12 (21.4)			*s1m2*	12 (14.6)	2 (2.9)	
***vacAm***	*m1*	40 (47.1)	46 (65.7)	0.020		*s2m1*	1 (1.2)	16 (23.2)	
	*m2*	45 (52.9)	24 (34.3)			*s2m2*	30 (36.6)	21 (30.4)	
***cagA***	+	74 (78.7)	42 (56)	0.002	***vacA sim***	*s1i1m1*	27 (39.7)	24 (45.3)	0.011
	-	20 (21.3)	33 (44)			*s2i2m2*	26 (38.2)	8 (15.1)	
						other	15 (22.1)	21 (39.6)	

**Table 5:** Distribution of *vacA* and *cagA* combination of *H*. *pylori* in duodenal ulcers and gastric cancer cases.

		N (%)							
		DU	GC	p-value			DU	GC	p-value
***vacAs/cagA***	*s1cagA+*	48 (54.5)	24 (33.3)	0.013	***vacA sm/cagA***	*s1m1cagA+*	34 (41.5)	22 (31.9)	0.005
	*s1cagA-*	6 (6.8)	8 (11.1)			*s1m1cagA-*	5 (6.1)	8 (11.6)	
	*s2cagA+*	21 (23.9)	16 (22.2)			*s2m2cagA+*	18 (22)	11 (15.9)	
	*s2cagA-*	13 (14.8)	24 (33.3)			*s2m2cagA-*	12 (14.6)	10 (14.5)	
***vacA i/cagA***	*i1cagA+*	31 (41.3)	26 (46.4)	>0.001		*other vacA/cagA+*	13 (15.9)	7 (10.1)	
	*i1cagA-*	4 (5.3)	18 (32.1)			*other vacA/cagA-*	0	11 (15.9)	
	*i2cagA+*	27 (36)	7 (12.5)		***vacA mi/cagA***	*m1i1/cagA+*	24 (34.3)	22 (40.7)	> 0.001
	*i2cagA-*	13 (17.3)	5 (8.9)			*m1i1/cagA-*	3 (4.3)	11 (20.4)	
***vacAm/cagA***	*m1cagA+*	35 (41.2)	27 (38.6)	0.002		*m2i2/cagA+*	24 (34.3)	7 (13)	
	*m1cagA-*	5 (5.9)	19 (27.1)			*m2i2/cagA-*	12 (17.1)	3 (5.6)	
	*m2cagA+*	32 (37.6)	14 (20)			*other cagA+*	6 (8.6)	4 (7.4)	
	*m2cagA-*	13 (15.3)	10 (14.3)			*other/cagA-*	1 (1.4)	7 (13)	
***vacA si/cagA***	*s1i1/casgA+*	27 (38)	18 (33.3)	0.004	***vacA sim/cagA***	*s1i1m1cagA+*	24 (35.3)	17 (32.1)	0.001
	*s1i1/cagA-*	3 (4.2)	7 (13)			*s1i1m1/cagA-*	3 (4.4)	7 (13.2)	
	*s2i2/cagA+*	15 (21.1)	5 (9.3)			*s2i2m2/cagA+*	15 (22.1)	5 (9.4)	
	*s2i2/cagA-*	12 (16.9)	5 (9.3)			*s2i2m2/cagA-*	11 (16.2)	3 (5.7)	
	*other/cagA+*	13 (18.3)	9 (16.7)			other *vacA/cagA+*	14 (20.6)	10 (18.9)	
	*other/cagA-*	1 (1.4)	10 (18.5)			other *vacA/cagA-*	1 (1.5)	11 (20.8)	

Thereafter, logistic regression analysis was performed and the results show that *vacA i1m1* and *vacA i1m2* genotypes were strongly associated with the risk of GC, the Odds Ratio (95% confidence interval) was 29.73 [5.08–173.73]) and 9.17 [2.06–40.82] respectively while vacA*s1/cagA*+ seem to be more associated to DU since it’s less present in GC cases, (OR: 0.13 [0.02–0.75] when compared with DU group “Table 6”.

**Table 6:** Risk for GC in relation to vacAs/cagA combination and i- and m-region of *vacA* gene in a simple logistic regression analysis.

		Frequency (%)		Simple logistic regression		
		DU	GC	OR	95% IC	p-value
*vacAs/cagA*	*s1/cagA+*	48 (54.5)	24 (33.3)	0.06	[0.01–0.37]	0.002
	*s1/cagA-*	6 (6.8)	8 (11.1)	0.19	[0.02–1.80]	0.152
	*s2/cagA+*	21 (23.9)	16 (22.2)	0.78	[0.21–2.84]	0.711
	*s2/cagA-*	13 (14.8)	24 (33.3)	1 (ref)	1 (ref)	
*vacA im*	*i1m1*	27 (38.6)	33 (61.1)	29.73	[5.08–173.73]	< 0.001
	*i2m1*	2 (2.9)	2 (3.7)	9.84	[0.66–145.84]	0.096
	*i1m2*	5 (7.1)	9 (16.7)	9.17	[2.06–40.82]	0.004
	*i2m2*	36 (51.4)	10 (18.5)	1 (ref)	1 (ref)	

## Discussion

The *H*. *pylori* infection is strongly associated with peptic ulcer disease (PUD), gastric carcinoma, and gastric mucosa-associated lymphoid tissue (MALT) [15]. This bacterium is known for its high genetic diversity that occurs mainly in the bacterium virulence factors as the cytotoxin-associated gene (*cagA*) and the vacuolating cytotoxin (*vacA*) gene. The variability that affects these two genes may be useful to better understand the differences in the pathogenesis and the role of each genotype in the occurrence of the pathology. In fact, polymorphisms within *vacA* have been analyzed in several previous studies, but they have primarily focused on the signal region (s region) and the mid-region (m region), both of which have been used to predict the clinical outcome of infected patients. In Morocco, we have previously studied the polymorphism of *vacA* in those two regions (signal sequences (s) and mid-region (m)) and established their correlation to clinical outcomes and also to histological lesions[1,10]. Of note, in this study, we confirmed that the studied population was predominantly infected by the less virulent *H*. *pylori* strains (*vacA s2m2*) and we confirmed a significant association between *vacA m1* and GC. However, it will be for interest to study *vacA i* alone and in combination to *cagA* status and to s and m *vacA* region in a large sampling to better characterize isolates that may lead to severe diseases, mainly the GC.

*H*. *pylori* is known to colonize more than half of the world’s population but the infection rate varies across geographic regions. Its prevalence differs by age, race, and socioeconomic level. In this series, *H*. *pylori* infection prevalence was 59.4%, this rate is comparable to our previous study (59.7%) [1] and to other developing countries but it is higher than in developed ones [16].

It is well known that almost all *H*. *pylori* strains contain the *vacA* gene, likewise in the current study we found it in 99.06% of cases. This rate is higher than those reported from Ethiopia (90%) and Netherlands (93%) [9,17]. The failure on *vacA* gene detection in 0.94% cases can be due to the genomic diversity of the bacteria which can be due to point mutations in the conserved genes. In fact, the multiple infection contribute to the genetic diversity by the extensive inter strain gene transfer and recombination that include large insertions or deletions and chromosomal rearrangements [18]. In this study, the presence of multiple *H*. *pylori* strains in a single biopsy specimen was found in 14.2% of cases. This was similar to the results obtained in Senegal (14%), Netherlands (11%), North of America (11%), Brazil (13%), India (15.9%) and Spain (19.2%) [7,19–22], and it was lower compared to the reported results in Tunisia (31.4%) Chile (32%) and Portugal (37.3%) [23–25]. The presence of multiple infections in developed countries as in the developing countries is totally inconsistent with the idea that mixed infections predominate only in the developing countries by the fact that it can be related to the high prevalence of *H*. *pylori* [26].

The polymorphism in the *vacA* gene sequence has been studied in three variable regions, and the *vacA* genotyping revealed the predominance of *vacA s2*, *vacA i2* and *vacA m2* alleles which were present in 52.9%, 52% and 53.2% of the cases respectively (Table 1). This distribution is almost similar to the follow-up study conducted on Spanish population [7,27] where these alleles (*vacA s2*, *vacA i2* and *vacA m2)* were present in 53.6%, 55.7% and 52%, and to the epidemiological study conducted on Kenyan population [28], where the *vacA s2*, *vacA i2* and *vacA m2* alleles were found at 49%, 52% and 65%, respectively. However, it’s totally different from the profile reported in Bulgaria and Senegal, where *vacA s1*, *vacA i1* and *vacA m1* alleles were predominant [8,29]. The results are also different from those obtained in Vietnam and marked by the predominance of *vacA s1*, *vacA i1* and *vacA m2* alleles [30].Those results show a great diversity of the bacterium.

All possible combinations of the three *vacA* regions “Table 1” have been reported, showing a high genetic diversity in our *H*. *pylori* clinical isolates. Excluding the multiple infection and the incomplete *vacA*, *vacA* genotype distribution reveals a large predominance of *vacA s2i2m2* with a rate of 44.3% (n = 211), while *vacA s1i1m1*, *vacA s1i2m2*, *vacA s2i1m2 and vacA s2i1m1* were detected in 28.8% (n = 137), 8% (n = 38), 5.3% (n = 25) and 4.6% (n = 22) of cases respectively. Unfortunately, there is no data from the North African countries regarding the polymorphism of the three region of *vacA*. In our study, the determined distribution is almost similar to the one determined among Sicilian and Kenyan patients [28,31] and differs from the others reported among European, Senegalese, Iranian, Malaysian and the South Korean ones, where *vacAs1i1m1* was the most predominant genotype [8,32–34], while *vacA s1i2m2* was the prevalent genotype in Iranian population [35]. This difference may explain the predominance of severe diseases in these regions. In effect, it is well established that the countries with high rates of severe diseases have strains carrying the *vacA* active forms [5]. This was confirmed by our moderate rate of *vacA s1i1m1* strains in the studied population related to the lowest rate of the serious diseases like gastric cancer.

In the present study, the distribution of the intermediate region of *vacA* among the s and m regions reveals that 89% and 89.4% of *vacA s1m1* and *vacA s2m2* strains were *vacA i1* and *vacA i2* respectively (Table 2), which is similar to the results of alleles association in most studies [5,8,36]. Also, our results show that 29.62% of the *vacA s1m2* strains were *vacA i1*, when it was 66.7% in Uruguayan, 92% in Chinese, 71.4% in U.S.African strains and 66.7% in East Asian [36,37]. This might be one hypothesis to explain the African enigma, since as it was approached by Rhead and Coll, strains of *vacA s1i1m2* genotype are more virulent, and more associated with GC than the *vacA s1i2m2* genotype [5].

The prevalence of *H*. *pylori cagA+* (61.2%) confirm the data obtained in our previous study (59.6%)on smaller sampling [1].

Our study also demonstrates that *vacA i1* was strongly associated with the *vacA s1*, *vacA m1* and *cag*A+ genotypes. As well, *vacA i2* was strongly associated with the *vacA s2*, *vacA m2* and *cagA-* genotypes (p<0.001), suggesting that the polymorphism of *vacA* gene may affect the functional interaction with *cagA* [38]. Those results are in agreement with numerous data from Chinese, U.S. African, Uruguayan, Iranian, Iraqi, Jordanian and Turkish studies reporting that *H*. *pylori* strains containing the active *vacA* gene (*s1i1m1*) carry also *cagA* gene, and *H*. *pylori* strains that carry the inactive *vacA* gene (*s2i2m2*) lack the *cagA* gene [5,37,39–41]; this clustering of active virulence factors within *H*. *pylori* strains classified *H*. *pylori* to be virulent or nonvirulent.

In this series, *vacA* genotypes were significantly associated to the patient’s gender. Effectively, the rate of *vacA s1i1m1* was higher in men than in women while the rate of *vacA s2i2m2* was higher in women than in men (p = 0.009). This finding confirms that men are more susceptible to develop severe disease than women; these results are confirmed in the present study in which GC, GU, and DU were predominantly present in men (63%, 69.7% and 73.7% cases respectively). These results confirmed those reported in Bulgarian patients, who have reported a high rate of strains containing the less virulent genotype (*vacA s1i2m2*) in women than in men [29].

In our results, *cagA* is significantly associated with age and is predominant in younger patients (67.3%) vs (54.5%) older ones (p = 0.001). And despite the absence of significant difference in *vacA* genotypes distribution according to the age, we noted that in contrary to *vacA i1* allele, *vacA s1* and *vacA m1* are increased slightly in age group 1 than in age group 2 which is in disagreement with the work of Alarcón and his colleagues who report that the prevalence of *cagA*, *vacA s1*, *vacA i1* and *vacA m1* were more frequent in older patients than in younger [42]. This raises the hypothesis on the evolution of *vacA H*. *pylori* genes in our population towards virulent form, presumably, due to adaptation of the bacteria to the host immune responses and to the environmental change. In fact, Gangwer *et al*., supposed in 2010 that *H*. *pylori cagA* and *vacA* can evolved separately from the bacteria core genome [43].

The results show also that patients with severe diseases (GU, DU and GC) are commonly infected with *vacA s1*, *vacA m1* and *vacA i1* strains (except of patients with gastric cancer who are frequently infected with *vacA s2*, *vacA m1* and *vacA i1* alleles) in contrast to patients with gastritis who are commonly infected with *vacA s2*, *vacA m2* and *vacA i2* strains (Table 3). Similar results were found in Western populations, while in East Asian population, *vacA s1*, *vacA m1* and *vacA i1* alleles were common in both dyspeptic and non-dyspeptic patients. In this regard, the profile of our strains and their association to the pathologies are similar to those isolated in Western populations, since the active form of *vacA* is not so prevalent and is more frequently found in ulcer or gastric cancer patients from Western populations, conversely, in the Eastern populations, these genotypes has been detected in almost all *H*. *pylori* strains. This finding will be confirmed by the studying of *cagA* EPIYA motifs polymorphism.

Since gastritis can evolved towards GU which predispose to GC disease, and since DU and GC seem to be mutually exclusive outcomes of *H*. *pylori* infection [44], only this two pathologies were used in statistical analysis to determine their risk factors. In univariate analysis, *vacA s2* allele (the least virulent) is significantly associated with gastric cancer (Table 4). Also high prevalent of this allele was reported in GC cases in our previous study [10]. Likewise, the less virulent genotypes (*vacA s2*, *vacA m2* and *vacA s2m2)* were reported in patients with MALT lymphoma among Mexican, Germany and French populations respectively [45–47]. Those results are in disagreement with several previous studies that report a high rate of the virulent alleles (*vacA s1*, *vacA m1*, and *vacA i1*) in patient with sever diseases [48–50]. Indeed, *vacA s2* genotype encodes a short extension of the N-terminal peptide on the mature protein, which blocks the vacuolating activity [51]. So, the occurrence of *vacA s2* in GC lets suppose that: i) the possible involvement of other factors including human genetic polymorphisms and diet in the gastric cancer genesis [10] ii) a high bacterial load in patients is a factor of gastric cancer genesis independently of the bacterial genotype. In effect, some studies reported an association between an high rate of the bacterium and the development of severe diseases independently to their virulence factors [52,53].

*H*. *pylori* infection is an etiological factor common to GC and DU diseases and may be one of the major causative factors of those opposite end outcome of the infection. So it is practical to find the *H*. *pylori* genotypes associated to each disease. Despite the common distribution of the active form of *vacA* and *cagA* genes among different *H*. *pylori* isolates in patients with DU and GC and the difficulty to distinguish *H*. *pylori* strains associated to GC from those associated to DU in the previous studies, the regression analysis results of the present study (Table 6) shows that:

- Patients infected with *vacA i1m1* and *vacA i1m2* have a significant 29.73 fold (95%, CI, 5.05–173.73) and 9.17 fold (95%, CI, 2.06–40.82) respectively increased risk of GC in comparison with those infected with *vacA i2m2* strains. This significant association let’s suppose that *vacA i* is more important in predicting the risk of GC in our series. This result support those reported in Iran, Spain, United States and Colombia [5,27,32,37,54] and is inconsistent with studies that report the non usefulness of this gene as a risk factor for GC [55–57].
- The infected patients with strains carrying the *vacA s1* allele and *cagA* gene have a greater probability of developing DU than GC since we have compared GC with DU. This finding supports the role of *vacA s1* and *cagA+* in the development of ulceration, confirming previous studies in western countries [3,19]. However, a study conducted on Portuguese patients reported the associations of this genotypes with both of GC and DU diseases [25].

These results suggest the presence of separate pathways to discriminate the risk of GC development from DU and suggest that *H*. *pylori* genotypes may play an important role in the infection outcomes. However, the apparent discrepancy between our results and those reported in other studies could be related to geographic variations between *H*. *pylori* strains, or probably by differences in the population origins or inflammatory responses governed by host genetics. Overall, the association between virulence factors and disease outcomes can be hampered by the difference in food habits and living conditions in each region as well as by dissimilarity of the compared gastric diseases. In fact, none of the previous studies has compared the GC with DU but they used either nonulcer disease or chronic gastritis as control groups. Those groups could be evolved towards GC or DU, inversely DU and GG seems to be two “mutually exclusive” end outcomes of *H*. *pylori* infection [44]. Indeed, conclusions data derived from a single geographic region will not be considered as representative of what will be found in other geographical regions.

## Conclusions

This study indicates that: i) *H*. *pylori* strains isolated from Moroccan patients are extremely diverse, ii) the less virulent strain *vacA s2i2m2* is the most predominant in Morocco which can partially explain the African enigma, iii) vacA*s1/cagA*+ seems to be a risk factor for DU, iv) *vacA i1* genotype (independently to *vacA m* status) isstrongly associated to the GC. So, patients infected with this genotype may need more attention. Therefore, *vacA i1* may be of a clinical usefulness to identify patients at a high risk of GC development.
